# Crossed Erythrocytes Agglutination Pattern Observed in a Patient With Hereditary Elliptocytosis

**DOI:** 10.1111/ijlh.70054

**Published:** 2025-12-30

**Authors:** Márcio A. W. Melo, Cristina M. Silveira, Maíra M. Ribeiro, Gabriela S. Arcanjo

**Affiliations:** ^1^ University of Pernambuco Recife Pernambuco Brazil; ^2^ Laboratório Marcelo Magalhães Recife Pernambuco Brazil; ^3^ Hospital Alcides Carneiro Recife Pernambuco Brazil; ^4^ Genetics Postgraduate Program Federal University of Pernambuco Recife Pernambuco Brazil

## 1 Case Image

Hereditary elliptocytoses (HE) are a group of autosomal dominant disorders characterized by peripheral blood smears showing at least 70% of erythrocytes with an elliptical shape. This abnormal morphology occurs because mutations in the spectrin protein of the cytoplasmic membrane cause erythrocytes to lose elasticity. Defects in spectrin dimer–dimer associations and in the spectrin–actin–protein 4.1 complex of the red cell membrane junction lead to weaker horizontal connections in the cytoskeleton. HE affects populations worldwide, with an estimated incidence of approximately 1 in every 2.000 to 4.000 individuals. It is more prevalent in malaria‐endemic areas, as elliptocytes may offer some resistance to the protozoan, providing a selective advantage to those affected with HE. For example, in West Africa, the prevalence of HE is approximately 1 in every 100 individuals [1].

**FIGURE 1:**
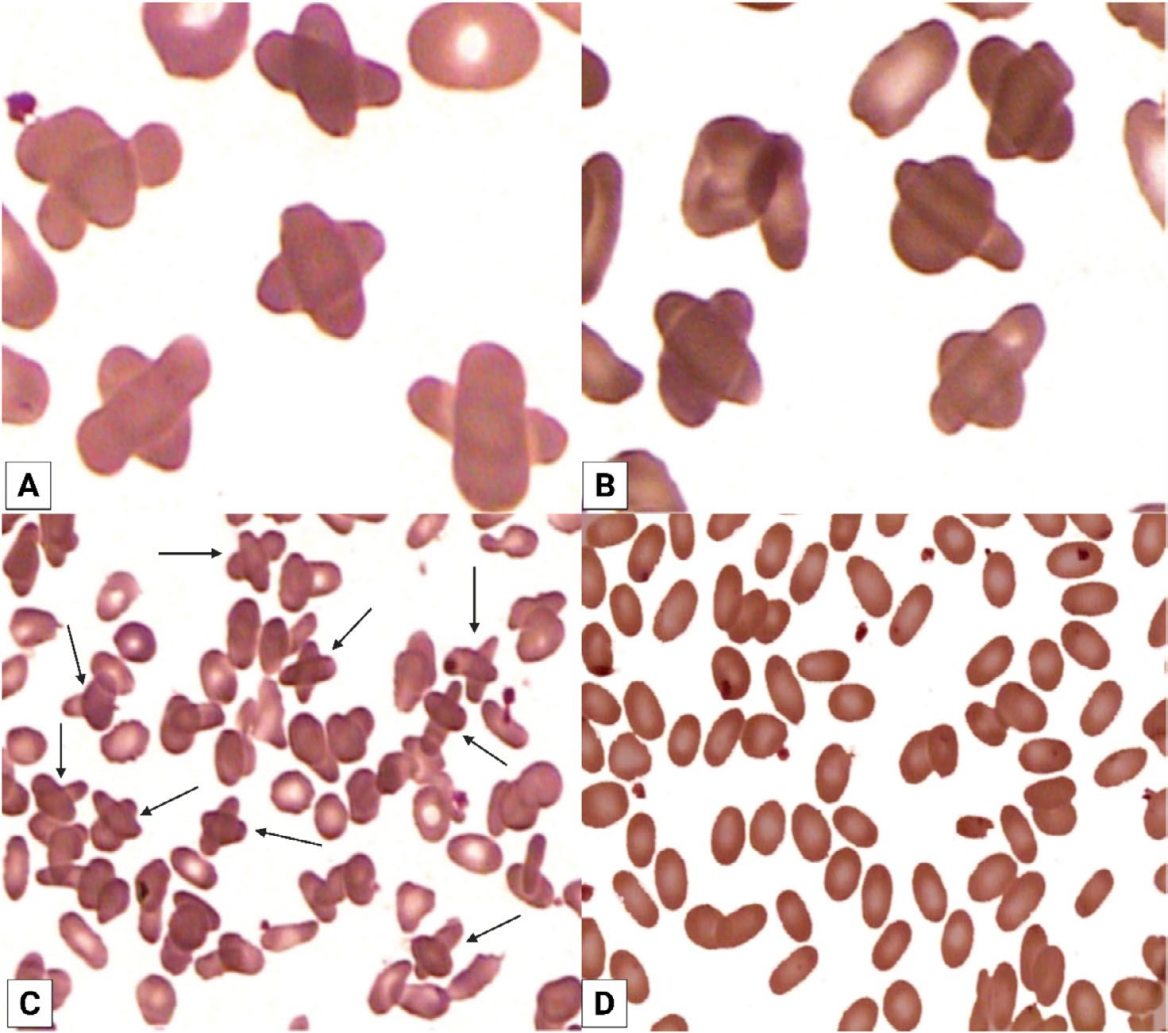
Peripheral blood smear stained with May‐Grünwald‐Giemsa. (A, B) Magnification 2000×, showing multiple “crossed” erythrocytes. (C) Magnification 800×, also demonstrating the presence of several “crossed” erythrocytes. (D) After washing the whole blood five times with 0.9% saline, the peripheral blood smear (800×) shows the absence of “crossed” erythrocytes, indicating that the phenomenon was reversed by restoring normal electrical charge between erythrocytes.

Here, we report unusual findings in an 88‐year‐old woman with the symptomatic form of HE and no family history of erythrocyte membrane disorders. The patient did not report any medication use, nor were there signs of hepatic, renal, or endocrine dysfunction. Her blood tests showed a normal leukocyte count (7.9 × 10^9^/L; reference: 4–11 × 10^9^/L) and a platelet count (166 × 10^9^/L; reference: 150–450 × 10^9^/L). However, her hematimetric indices revealed anemia, with a low erythrocyte count (2.4 × 10^12^/L; reference: 4.0–5.4 × 10^12^/L), hemoglobin level (7.4 g/dL; reference: 12.0–16.0 g/dL), and hematocrit (21.9%; reference: 36%–46%). The red blood cell indices were as follows: mean corpuscular volume (MCV) 91.2 fL (reference: 80–101 fL), mean corpuscular hemoglobin (MCH) 30.8 pg (reference: 27–33 pg), mean corpuscular hemoglobin concentration (MCHC) 33.7 g/dL (reference: 31.5–36 g/dL), and red cell distribution width (RDW) 16.3% (reference: 11.5%–15.5%), indicating anisocytosis. Her peripheral blood smear showed over 75% elliptocytes and the peculiar presence of erythrocytes that agglutinated and were arranged perpendicularly to each other, forming a cross or an “*X*” shape (Figure 1A–C).

To rule out other hematological disorders that could explain this unusual erythrocyte arrangement, additional tests were performed. A myelogram presented normal results (apart from the presence of elliptocytes), and immunophenotyping of peripheral blood also showed no abnormalities. Together, these findings confirmed the diagnosis of HE.

To date, erythrocyte agglutination has only been described in the form of *rouleaux*—where red cells stack on top of one another—or in cases triggered by cold or warm antibodies, which result in irregular aggregates of erythrocytes [2]. However, “crossed” erythrocytes have not been described in either of these conditions.

To investigate the cause of these crossed erythrocytes, a sample of whole blood was washed five times with 0.9% saline solution. New blood smears were then prepared. The cytological assay showed that the crossed erythrocytes disappeared (Figure 1D), suggesting that their formation was due to alterations in surface electric charges between erythrocytes, which were reversed by the saline washes [3]. Three months later, a follow‐up blood smear confirmed the recurrence of “crossed” erythrocytes, and again, saline washing abolished their formation.

Interestingly, the presence of “crossed” erythrocytes has also been occasionally observed by our group in the peripheral blood of other HE patients.

## Author Contributions

M.A.W.M., C.M.S., and M.M.R. wrote the manuscript and contributed to the diagnosis of the case. G.S.A. critically revised the article. All authors read and approved the final version of the manuscript.

## Funding

The article processing charge for the publication of this research was funded by the Coordenação de Aperfeiçoamento de Pessoal de Nível Superior ‐Brasil (CAPES).

## Ethics Statement

The authors have nothing to report.

## Consent

The authors have nothing to report.

## Conflicts of Interest

The authors declare no conflicts of interest.

## Data Availability

The data that support the findings of this study are available from the corresponding author upon reasonable request.
